# The Effect of Music Distraction on Dental Anxiety During Invasive Dental Procedures in Children and Adults: A Meta-Analysis

**DOI:** 10.3390/jcm13216491

**Published:** 2024-10-29

**Authors:** Kung-Chien Shih, Wei-Ti Hsu, Jia-Li Yang, Kee-Ming Man, Kuen-Bao Chen, Wei-Yong Lin

**Affiliations:** 1 Department of Anesthesiology, China Medical University Hospital, Taichung 404327, Taiwanu108305203@cmu.edu.tw (W.-T.H.); 028493@tool.caaumed.org.tw (J.-L.Y.);; 2 Graduate Institute of Integrated Medicine, China Medical University, Taichung 404328, Taiwan; 3 Department of Anesthesiology, China Medical University Hsinchu Hospital, Hsinchu 302056, Taiwan; 4 Department of Anesthesiology, College of Medicine, China Medical University, Taichung 404328, Taiwan; 5 Department of Medical Research, China Medical University Hospital, Taichung 404327, Taiwan

**Keywords:** music distraction, dental anxiety, meta-analysis

## Abstract

**Background:** Dental anxiety and odontophobia are common issues, leading to challenges with oral hygiene and dental health. Music distraction offers an effective and side effect-free solution to alleviate pain and increase the acceptability of dental treatments. Our meta-analysis aimed to assess the efficacy of music distraction in reducing patient anxiety during invasive dental procedures in children and adults. **Methods:** The PubMed, Web of Science, and Embase databases were searched for clinically controlled trials, using the keywords “music” and “dental anxiety”. The main outcome measured was the anxiety score. A meta-analysis was conducted using a random-effects model to estimate the standardized mean differences (SMDs). The subgroup analyses were conducted based on age groups, music preferences, and music styles. The research protocol has been registered with PROSPERO (Registration ID: CRD42022357961). **Results:** A total of 24 controlled clinical trials involving 1830 participants met the inclusion criteria for the meta-analysis. Music distraction significantly reduced dental anxiety during invasive procedures under local anesthesia (SMD, −0.50; 95% CI, −0.80 to −0.21; *p* = 0.0009; *I*^2^ = 83%). Our subgroup analysis revealed that music distraction was more effective in adults (SMD, −0.51; *p* = 0.0007) than in children (SMD, −0.47; *p* = 0.13) in reducing dental anxiety. Regarding music selection, music chosen by the participant (SMD, −1.01; *p* = 0.008) demonstrated more anxiolytic effects than by the author (SMD, −0.24; *p* = 0.02). Regarding music styles, classical music (SMD, −0.69; *p* = 0.009) was associated with better anxiolytic effects in adults. **Conclusions:** Our meta-analysis supports the use of music to alleviate dental anxiety during invasive procedures. Listening to classical or customized music can serve as an effective adjunct to outpatient surgical care in dental clinics.

## 1. Introduction

Common dental diseases, such as dental caries, tooth loss, and periodontal disease, affect more than 3 billion people worldwide [1]. Untreated dental diseases may lead to imbalanced nutrition, growth and developmental problems, malocclusion, social phobia, and even serious heart infections [2,3,4,5]. In Taiwan, the prevalence of untreated caries in permanent teeth is 29.0% in children and 33.6% in adults. At the same time, up to 78.7% of adults experience periodontal diseases [6,7]. Dental fear, also known as dental anxiety or dental phobia, is a significant contributor to the high prevalence of dental diseases [8]. Individuals with dental anxiety often avoid dental visits, which can worsen their dental problems over time.

Dental anxiety is a negative emotional reaction to dental treatments, leading patients with dental problems to worry about unpredictable and uncontrollable situations that may occur during treatment procedures [9]. Globally, approximately 30% of young children and 15% of adults experience dental fear and anxiety [10,11]. Adults with dental fear may experience anxiety due to various factors, such as the noise made by dental instruments, odors of blood and filling materials, fear of gagging, choking or breathing difficulties from a stuffed oral cavity, and apprehension about numbness and pain following local anesthetic injections or invasive dental procedures. Children with dental fear may feel anxious about the unfamiliar staff and environment of the dentist’s office, the incomprehensible procedures, and the potential embarrassment over or punishment for a poor dental report [12]. Dentists and clinic staff have implemented various strategies to help patients overcome their fears, including pre-clinic communication and education, friendly clinic decoration, relaxing music, fun audible stories, movies, and even video games with high-tech virtual reality headsets [13].

The efficacy of music distraction in alleviating dental anxiety in children and adults has been validated by many clinical studies and meta-analyses [14,15]. Most dental treatments discussed in the literature are noninvasive procedures, such as dental prophylaxis and tooth restoration. However, patients undergoing more invasive procedures, such as tooth extractions and dental implants, tend to experience heightened anxiety. There is a lack of evidence regarding the effectiveness of music as a distraction during these invasive treatments. Hence, our study aimed to assess the efficacy of music in alleviating dental anxiety for children and adults, specifically during invasive dental procedures. To achieve this, we conducted a meta-analysis of relevant clinical trials to assess the impact of music distraction on dental anxiety.

## 2. Materials and Methods

This meta-analysis was conducted following the Preferred Reporting Items for Systematic Reviews and Meta-Analyses (PRISMA) guidelines (Supplementary Table S1) [16]. The protocol for this meta-analysis has been registered on the International Prospective Register of Systematic Reviews (CRD42022357961) at https://www.crd.york.ac.uk/prospero/display_record.php?ID=CRD42022357961, accessed on 20 October 2022.

### 2.1. Search Strategy

Two authors, K.-C. Shih and J.-L. Yang, independently searched three popular electronic databases (PubMed, Web of Science, and Embase) for clinical trials on the impact of music distraction on dental anxiety from the inception date to May 2024. They also searched for relevant review articles for further studies. The search terms used were “music” and “dental anxiety”. In PubMed, the search strings included (music OR song OR audio) AND (dental OR tooth) AND (anxiety).

Two independent authors, K.-C. Shih and J.-L. Yang, assessed the title, abstract, and full text of all identified articles for eligibility based on the following inclusion criteria: (1) parallel-group controlled clinical trials in humans; (2) participants who underwent dental procedures; (3) participants who younger than 12 years old or older than 18 years old; (4) the intervention used during the dental procedures; (5) outcome assessment, including anxiety scores throughout the dental procedures; and (6) publications written in English. The exclusion criteria were as follows: (1) review articles, protocols, conference papers, case reports, letters, and editorials; (2) use of music combined with other interventions for distraction; (3) control group with any audio intervention; (4) insufficient data for analysis; (5) inability to obtain full text from a website; and (6) use of the same study population as other works from the literature. Any discrepancies were resolved by consulting the advising professor, W.-Y. Lin.

### 2.2. Data Extraction

Two authors, W.-T. Hsu and K.-M. Man, independently extracted clinical information and data from all the included studies. The clinical information included the following details: (1) characteristics of the papers (authors, publication year, country, and study design); (2) characteristics of the participants (study groups, sample size, mean age, types of dental procedures, and with or without local anesthesia); (3) music interventions (music style and music selection); and (4) outcome measures with statistical data. The primary outcome was the anxiety score, mainly based on self-report or objective assessment. If a study included multiple anxiety scores, the Venham Picture Test, State–Trait Anxiety Inventory, and Facial Image Scale were preferred for meta-analyses. Data were extracted mainly from post-intervention measurements or change-from-baseline measurements if means and standard deviations were available. In cases of incomplete public data, the relevant literature was consulted, or authors were emailed for supplementary data. Since all analysis data were extracted from the public literature, Institutional Review Board approval was not required.

### 2.3. Methodological Quality Assessment

The methodological quality of all included studies was independently assessed by two authors, K.-M. Man and K.-B. Chen, using the modified Jadad scale [17]. The scale comprises eight items to evaluate randomization, blinding, withdrawals, dropouts, inclusion and exclusion criteria, adverse effects, and statistical analysis. The total score ranged from 0 to 8, with a score of 7 or 8 indicating high quality, 4–6 indicating moderate quality, and 1–3 indicating low quality. The certainty of the evidence for the main outcomes was also assessed by the authors K.-C. Shih and W.-T. Hsu, using the Grading of Recommendations Assessment, Development and Evaluation (GRADE) methodology [18]. The GRADE assessment items included risk of bias, inconsistency, indirectness, imprecision, other issues, and effect size. The quality of the evidence ranged from very low to high. Any disagreements were resolved by consulting the advising professor, W.-Y. Lin.

### 2.4. Statistical Analysis

All statistical analyses and meta-analyses were conducted using Review Manager 5 software (version 5.4, The Nordic Cochrane Center, Copenhagen, Denmark). Pooled effect estimates (ESs) were calculated by determining the standard mean difference (SMD) in outcomes for continuous variables, along with their respective 95% confidence intervals (CIs). Values less than zero indicated more effective outcomes in the music group. A *p*-value of less than 0.05 was considered significant for the analysis of ESs. Heterogeneity was assessed using I-square statistics (*I*^2^), with an *I*^2^ over 50% indicating substantial heterogeneity [19]. The random-effects model was used to estimate the pooled ESs. In studies with multiple music groups, groups were combined to create a single pair-wise comparison, or the “shared” group was split into two or more groups with smaller sample sizes [20].

Subgroup analyses were conducted based on age group (adults or children), music selection by participant preference or author, music style for children, and music style for adults. Afterward, a subgroup analysis was conducted to assess the impact of music and audiovisual interventions on anxiety at different stages of dental visits. Sensitivity analyses were conducted to assess the robustness of the findings by excluding non-randomized controlled trials or low-quality articles. Publication bias was assessed using a funnel plot [21], and the symmetry of the plot was visually inspected to identify potential publication bias. The GRADE assessment was used to assess the certainty of the evidence for the main outcome.

## 3. Results

### 3.1. Identification of Eligible Studies

**Figure 1:**
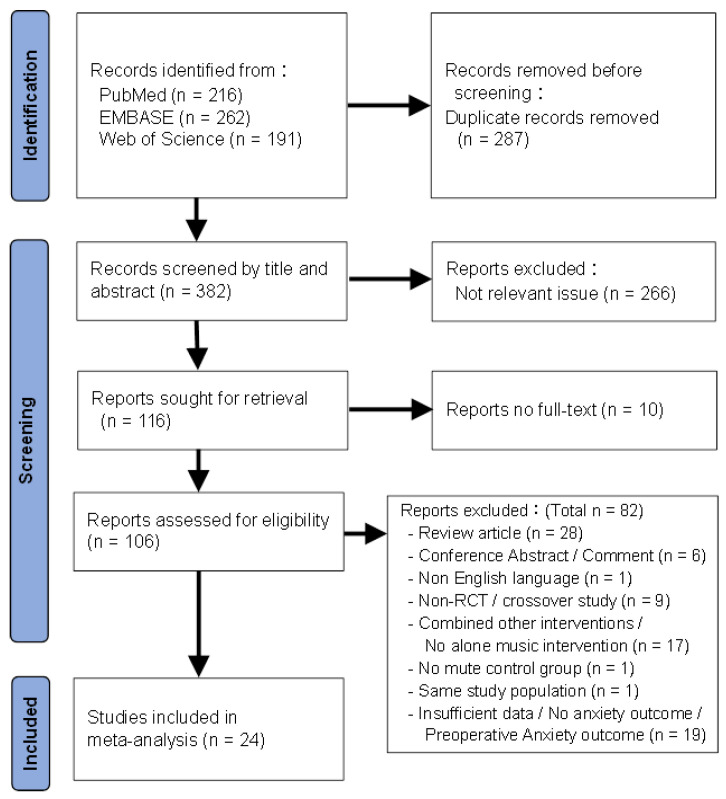
PRISMA flowchart of the selection strategy.

Figure 1 displays the result of our screening process. Initially, 669 articles were retrieved from three databases. After removing 287 duplicates and excluding 266 irrelevant articles, 24 out of 106 full-text articles meeting the selection criteria were included in the meta-analysis. The list of excluded full-text articles, along with the reasons for exclusion, is presented in Supplementary Table S2.

### 3.2. Study Characteristics

A total of 2335 participants from 24 articles published between 2002 and 2024 were included for analysis. The detailed characteristics of the included studies are presented in Table 1. Among the participants, 1525 received various forms of music distraction, audiovisual distraction, brief relaxation therapy, Bach flower therapy, or aromatherapy, while 810 received the control treatment. Among all studies, twelve were conducted in India [22,23,24,25,26,27,28,29,30,31,32,33]; three in Turkey [34,35,36]; two in Spain [37,38]; and one each in the USA [39], Germany [40], Taiwan [41], Korea [42], Japan [43], Pakistan [44], and Thailand [45]. Twenty studies followed a randomized controlled trial design [22,23,25,26,27,29,30,31,33,34,35,36,37,38,40,41,42,43,44,45], while four studies used a non-randomized controlled trial design [24,28,32,39]. The sample sizes ranged from 34 to 275 participants, with a mean age range of 3–57 years old. Specifically, 15 studies included children [22,23,24,25,26,27,28,29,30,31,32,33,39,44,45], and 9 studies included adults [34,35,36,37,38,40,41,42,43]. The dental procedures varied across studies, including oral prophylaxis, dental cleaning, restorative treatment, extraction, root canal treatment, pulp therapy, and dental implant surgery. Sixteen studies involved invasive dental procedures with local anesthesia [23,24,25,26,27,28,29,34,35,36,37,38,39,41,42,43]. The choice of music intervention was determined by the author in 15 studies [22,24,26,28,29,30,32,33,34,35,37,38,39,43,44] and by participant preference in 9 studies [23,25,27,31,36,40,41,42,45]. The type of music used was categorized into folk music [28,39], instrumental music [23,26,28,30,34,35,37,39,45], nursery music [23,26,44], popular music (movie songs, regional music, and Turkish music) [26,27,29,34,35,45], classical music [34,35,38,41,43], and special frequency music (monaural beats and binaural beats) [22,33]. Various anxiety measurement tools were used across studies, including the Venham Picture Test, modified Corah anxiety scale, North Carolina Behavior Rating Scale, Venham’s clinical anxiety rating scale, State–Trait Anxiety Inventory, hierarchical anxiety questionnaire, Corah’s dental anxiety scale, faces version of the modified child dental anxiety scale, Raghavendra, Madhuri, Sujata pictorial scale, modified dental anxiety scale, facial image scale, and Children’s Fear Survey Schedule—Dental Subscale.

### 3.3. Quality of the Included Articles

The modified Jadad scores for the methodological quality assessment of each selected study are illustrated in Table 2. Almost all studies did not have a double-blind design. Moreover, no studies assessed adverse effects of music intervention. Nine studies had low quality [23,24,25,26,28,29,32,34,35], and fifteen studies had moderate quality [22,27,30,31,33,36,37,38,39,40,41,42,43,44,45]. The average score of the included twenty-four studies was 4.1, which indicated overall moderate quality. We also utilized the Cochrane risk-of-bias tool to assess the risk of bias for all included studies (Supplementary Figure S1).

### 3.4. Main Outcomes of the Music Intervention

**Figure 2:**
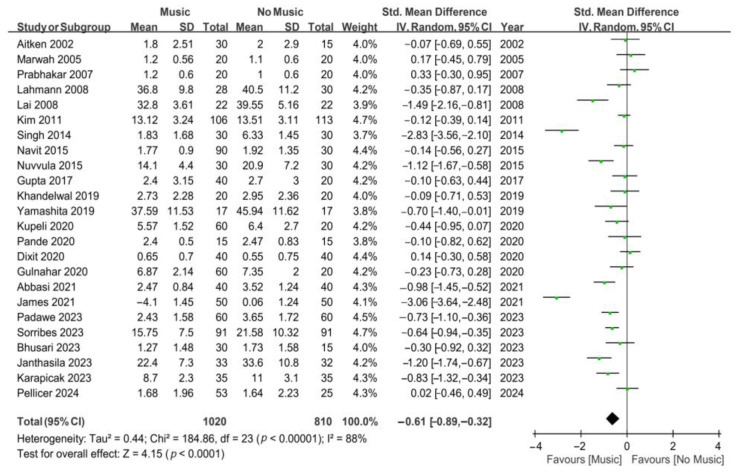
Forest plots for the effects of music on dental anxiety. James’s 2021 data represent the differences between pre- and post-treatment measurements, while the remaining data are all post-treatment measurements [22,23,24,25,26,27,28,29,30,31,32,33,34,35,36,37,38,39,40,41,42,43,44,45]. The green dots represent the effect sizes from individual studies, while the diamond shape rep-resents the overall summary effect size.

**Figure 3:**
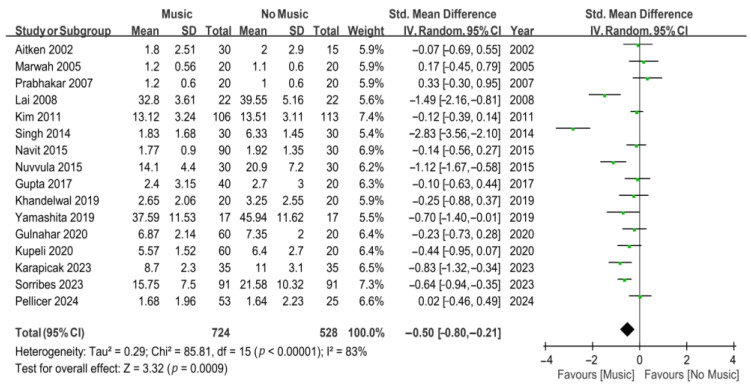
Forest plots for the effects of music on dental anxiety during invasive procedures [23,24,25,26,27,28,29,34,35,36,37,38,39,41,42,43]. The green dots represent the effect sizes from individual studies, while the diamond shape rep-resents the overall summary effect size.

In the meta-analysis of all 24 studies (Figure 2), the results revealed that music intervention significantly reduced anxiety compared to the control group (SMD, −0.61; 95% CI, from −0.89 to −0.32; *p* < 0.0001). However, substantial heterogeneity was noted (*I*^2^ = 88%). Another meta-analysis was conducted, including 16 studies on the effect of music intervention on anxiety in dental outpatients undergoing invasive procedures under local anesthesia (Figure 3). The results indicated that music intervention effectively alleviated dental anxiety during invasive procedures under local anesthesia compared to the control group, with substantial heterogeneity (SMD, −0.50; 95% CI, from −0.80 to −0.21; *p* = 0.0009; *I*^2^ = 83%).

### 3.5. Age Group

**Figure 4:**
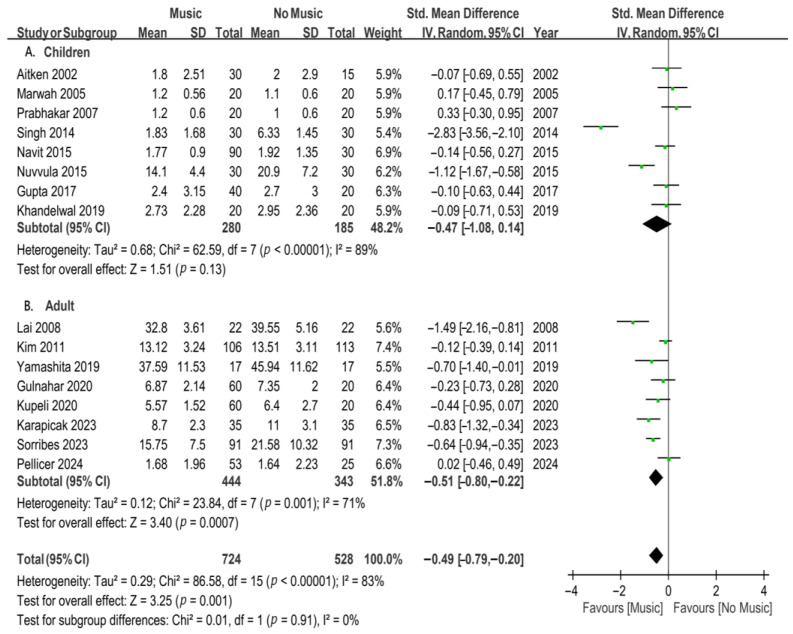
Forest plots for subgroup meta-analysis according to age group in patients undergoing invasive dental procedures [23,24,25,26,27,28,29,34,35,36,37,38,39,41,42,43]. A, children; and B, adult. The green dots represent the effect sizes from individual studies, while the diamond shape rep-resents the overall summary effect size.

The subgroup analysis was conducted based on age groups for participants undergoing invasive procedures under local anesthesia (Figure 4). Eight studies were included to analyze the impact of music on children. The results of the subgroup analysis indicated that music intervention did not significantly improve dental anxiety in children (SMD, −0.47; 95% CI, from −1.08 to 0.14; *p* = 0.13; *I*^2^ = 89%). Eight studies were included to analyze the effect of music on adults. The results of the subgroup analysis showed a significant reduction in dental anxiety among adults with music intervention (SMD, −0.51; 95% CI, from −0.80 to −0.22; *p* = 0.0007), but with substantial heterogeneity (*I*^2^ = 71%). A music intervention was found to improve dental anxiety in adults undergoing invasive procedures under local anesthesia.

### 3.6. Music Selected by the Participant or Author

**Figure 5:**
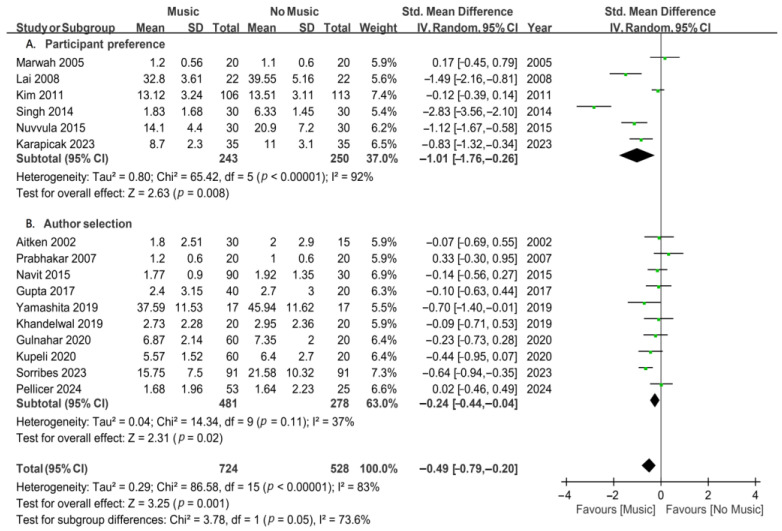
Forest plots for subgroup meta-analysis according to music selection during invasive dental procedures [23,24,25,26,27,28,29,34,35,36,37,38,39,41,42,43]. A, participant preference; and B, author selection. The green dots represent the effect sizes from individual studies, while the diamond shape represents the overall summary effect size.

Another subgroup analysis was conducted based on the differences in methods of music selection for participants undergoing invasive procedures under local anesthesia. The studies were divided into two groups: music selected by participant preference and music selected by the author (Figure 5). The subgroup analysis, including six studies, showed that music selected by participant preference significantly decreased anxiety in dental patients (SMD, −1.01; 95% CI, from −1.76 to −0.26; *p* = 0.008), but with substantial heterogeneity (*I*^2^ = 92%). The subgroup analysis, including 10 studies, showed that music selected by the author also significantly reduced anxiety in dental patients (SMD, −0.24; 95% CI, from −0.44 to −0.04; *p* = 0.02; *I*^2^ = 37%). Participant preference music was more effective in reducing dental anxiety than author-selection music. The results demonstrate that both participant-selected and author-selected music reduced anxiety in dental patients.

### 3.7. Types of Music for Children

**Figure 6:**
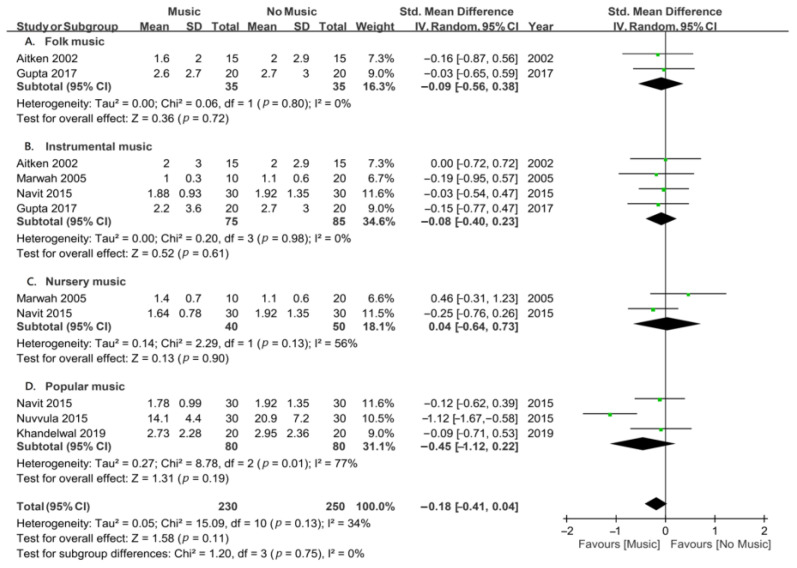
Forest plots for subgroup meta-analysis according to the types of music in children undergoing invasive dental procedures [23,26,27,28,29,39]. A, folk music; B, instrumental music; C, nursery music; and D, popular music. The green dots represent the effect sizes from individual studies, while the diamond shape represents the overall summary effect size.

Another subgroup analysis was conducted to investigate the therapeutic impact of music intervention on dental anxiety in pediatric participants undergoing invasive procedures under local anesthesia, focusing on various music genres. The analyses included four music genres: folk music, instrumental music, nursery music, and popular music (Figure 6). The results of the subgroup analyses revealed that none of the music genres significantly reduced dental anxiety in children: folk music in two studies (SMD, −0.09; *p* = 0.72); instrumental music in four studies (SMD, −0.08; *p* = 0.61); nursery music in two studies (SMD, −0.04; *p* = 0.90); and popular music in three studies (SMD, −0.45; *p* = 0.19). These findings suggest that differences in music genres had no specific impact on alleviating dental anxiety in pediatric participants undergoing invasive procedures under local anesthesia.

### 3.8. Types of Music for Adults

**Figure 7:**
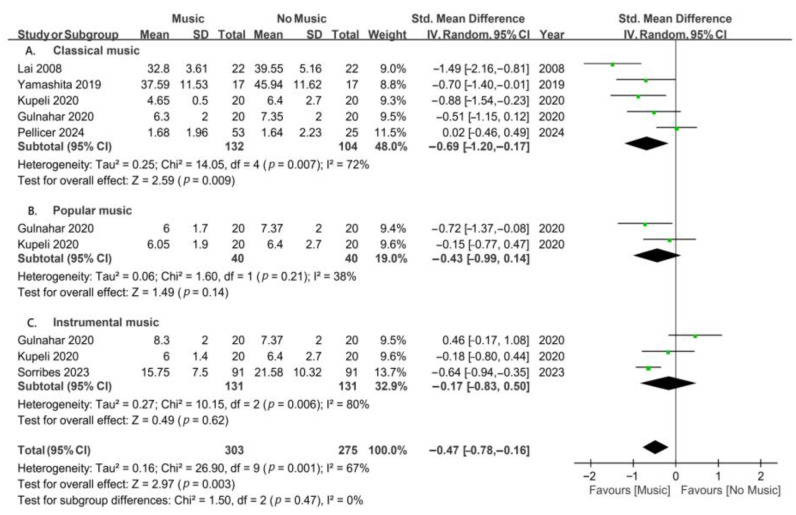
Forest plots for subgroup meta-analysis according to the types of music in adults undergoing invasive dental procedures [34,35,37,38,41,43]. A, classical music; B, popular music; and C, instrumental music. The green dots represent the effect sizes from individual studies, while the diamond shape represents the overall summary effect size.

In adults undergoing invasive procedures under local anesthesia, a subgroup analysis was conducted based on the types of music (classical, popular, and instrumental music; Figure 7). A subgroup analysis of five studies revealed that listening to classical music can significantly reduce dental anxiety in adults (SMD, −0.69; *p* = 0.009). However, a subgroup analysis of two studies revealed that listening to popular music did not significantly reduce dental anxiety in adults (SMD, −0.43; *p* = 0.14). Additionally, a subgroup analysis of three studies revealed that listening to instrumental music had no significant impact on reducing dental anxiety in adults (SMD, −0.17; *p* = 0.62). These findings suggest that classical music is more effective in alleviating dental anxiety in adults undergoing invasive procedures under local anesthesia.

### 3.9. Music Versus Audiovisual Interventions

Among the 24 articles reviewed, 3 studies in two articles (Prabhakar et al., 2007; and Khandelwal et al., 2019) [24,29] compared the effects of music and audiovisual (television) interventions on anxiety reduction during four dental visits. The first visit included screening and an intraoral examination. The second visit included oral prophylaxis. The third visit included cavity preparation, followed by restoration. The fourth visit included the administration of local anesthesia, followed by extraction or pulp therapy. The meta-analysis of these studies is presented in Table 3. At the first visit, a significant difference was found between the music and audiovisual groups (SMD, −0.44; *p* = 0.04) but not at the second (SMD, −0.37; *p* = 0.07) or third visits (SMD, 0.33; *p* = 0.11). However, at the fourth visit, a significant difference was observed (SMD, 0.70; *p* = 0.001). The findings suggest that audiovisual (television) intervention was more effective than music in alleviating anxiety in dental outpatients during screening and invasive treatments under local anesthesia. However, no significant difference was found for noninvasive procedures.

### 3.10. Sensitivity Analysis

Sensitivity analyses were conducted. First, only randomized controlled trials were included from all studies, and the results showed a significant effect (*k* = 20; SMD, −0.58; 95% CI, from −0.82 to −0.33; *p* < 0.0001). Second, by excluding the low-quality studies, the result of the sensitivity analysis showed a significant effect (*k* = 15; SMD, −0.56; 95% CI, from −0.79 to −0.32; *p* < 0.0001). Third, by excluding the study where anxiety scores were extracted from the change between pre- and post-treatment (James et al. 2021) [32], the result of the sensitivity analysis showed a significant effect (*k* = 23; SMD, −0.50; 95% CI, from −0.73 to −0.27; *p* < 0.0001). The sensitivity analyses all demonstrated a similar effect to the main analysis, indicating the robustness of the main results.

### 3.11. Evaluation of Publication Bias

**Figure 8:**
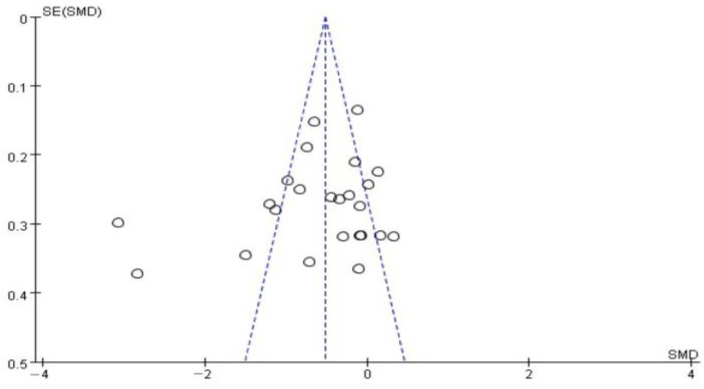
Funnel plot of standard difference, with means on the X-axis and standard error on the Y-axis for the effect of music on dental anxiety [22,23,24,25,26,27,28,29,30,31,32,33,34,35,36,37,38,39,40,41,42,43,44,45]. The circles represent individual studies.

The symmetry of the funnel plot of standard errors by SMD was assessed, and the results are depicted in Figure 8. The results revealed a symmetrical distribution of the funnel plot, indicating no publication bias in the 24 studies based on dental anxiety assessment [46]. The funnel plots for each outcome are presented in Supplementary Figure S2.

### 3.12. GRADE Assessment of the Evidence

The GRADE assessment is summarized in Table 4. Overall, the studies on dental anxiety were classified as having a low level of certainty due to the risk of bias and inconsistent estimates. Similarly, the studies on dental anxiety related to invasive procedures under local anesthesia were also classified as having a low level of certainty because of the risk of bias and inconsistent estimates. The GRADE assessment for all outcomes is presented in Supplementary Table S3.

## 4. Discussion

The clinical use of nonpharmacological treatments to reduce anxiety in patients during medical procedures has proven to be effective, particularly in dental settings [47,48,49]. Previous meta-analyses [50] have confirmed that music can effectively reduce anxiety during dental treatments. However, the literature has not conclusively demonstrated the utility of music for more invasive and painful dental procedures, such as tooth extractions [51,52,53]. This comprehensive meta-analysis indicates that listening to music during invasive dental procedures under local anesthesia considerably reduces anxiety compared with control groups who do not listen to music. Notably, music reduces anxiety in the waiting period before treatment [54,55] and during dental treatment procedures.

The impact of age on the effectiveness of music in reducing dental anxiety has garnered attention. Van der Weijden et al. reported that music effectively reduces dental anxiety in adolescents and adults [50]. In children, music has also been shown to reduce dental fear [56,57]. Invasive and complex dental procedures can increase pain and anxiety. Monteiro et al. reported that while music may help reduce preoperative anxiety in adults, it does not significantly impact physiological responses to perioperative pain [52]. Our subgroup analysis showed that music has a considerable effect on reducing anxiety levels in adults undergoing invasive dental procedures under local anesthesia. A previous meta-analysis showed that music can reduce dental and medical pain in children [58]. However, our analysis revealed that anxiety reduction is not significant in children. This discrepancy may be associated with the unclear expression of internal stress and emotions in children or the differences in anxiety assessment methods [9]. Therefore, further exploration of music’s effect on dental anxiety in children undergoing invasive procedures is warranted.

In addition, the subgroup analyses indicate that listening to personal favorite music or selected music can improve dental anxiety, with personally preferred music demonstrating a more significant effect. The literature has proven that listening to preferred music can better reduce anxiety or stress [59,60,61]. However, music chosen by the researchers, often characterized by specific melodies and rhythms, also provides a relaxing effect. Factors such as melody and rhythm are important in music’s ability to improve anxiety [62]. Ko CH et al. reported that light music is more effective than informal classical music in reducing anxiety during procedures like colonoscopies [63]. Kayaaltı-Yüksek S et al. reported that listening to Mozart’s music can reduce dental anxiety in children during tooth-brushing training [64]. Ting et al. reported that specific music styles, such as classical, kids, and pop music, have a considerable effect on reducing pain in children [58]. In our study, we conducted a subgroup analysis of different music styles, revealing that listening to classical music is particularly effective in calming anxiety in adults during invasive procedures under local anesthesia. Mozart’s sonata (K. 448) is frequently discussed in this context owing to its energetic and pleasant melody. Research suggests that Mozart’s music can influence arousal and mood states by altering neurophysiological activity in the brain, promoting relaxation, and enhancing cognitive abilities [65,66,67]. However, evidence regarding which music style is most effective in reducing dental anxiety in children remains insufficient. Therefore, when using music to relieve dental anxiety, it is crucial to consider personal preferences and select music with soothing melodies, rhythms, or sound frequencies. Furthermore, our analysis indicates that audiovisual distraction may be more effective than music alone in reducing dental anxiety during invasive procedures. Previous reviews have shown that audiovisual distractions can significantly improve anxiety and pain levels [68,69], effectively mitigating fear when music does not suffice.

Our meta-analysis had some limitations and recommendations for future research: (1) Most clinical studies lack direct comparisons between various music styles, necessitating further clinical trials to clarify the effects of specific music styles on dental anxiety. (2) Other sound interventions, such as storytelling or the sound of natural waves, wind, water, and artificially synthesized specific sound waves, were not included in the review, leaving their effectiveness unexamined. (3) The impact of listening to music via headphones versus speakers was not analyzed, preventing a comparison of their effects. (4) Most of the analysis results of this study are highly heterogeneous, which may be due to the melody/rhythm of various music affecting the listener differently from person to person. (5) The small number of included studies, or only articles published in English, raises the possibility for publication bias. (6) Most studies had sample sizes under 100, leading to low statistical power and increased variability. (7) The overall quality of the literature in this study was low to moderate, possibly due to the research model about listening to music. It is challenging to conduct double-blind research for the model of conscious people listening to music. Future clinical trials may further explore the effect of different music styles, sound types, and melody/rhythm/frequency on brain electrical activity and dental anxiety. In addition, future meta-analysis can include studies with larger sample sizes and high-quality clinical studies to further confirm the effectiveness of music on dental anxiety.

In pediatric dental clinics, unpleasant procedures are successfully completed using sedatives [70,71]. How to improve the safety of pediatric patients during the course of treatment with sedation has always been an important issue. If sedatives are used inappropriately in pediatric patients, conscious sedation may inadvertently change to a deep state, or even to a general anesthesia state, which may cause more serious complications [72]. Therefore, we recommend that clinicians consider music therapy in pediatric dental clinics: (1) Music-assisted therapy can help calm children’s anxiety and uneasiness and reduce the use of general anesthetics. (2) The choice of music should be based on the patient’s preference or Western classical music. (3) The method of listening to music is preferred because it is convenient and cost-effective option in outpatient settings. Moreover, audiovisual equipment can be used as an advanced or a second-line option. The use of music-assisted therapy may increase the safety of dental treatment and reduce the occurrence of complications.

## 5. Conclusions

This meta-analysis shows that music distraction can effectively alleviate dental anxiety associated with invasive and painful procedures. Given the difference in music preferences and styles, listening to patient-preferred music or classical music may be effective in calming dental anxiety. In addition, combining music with audiovisual interventions may enhance anxiety-reducing effects in dental clinical environments. These findings underscore the importance of music distraction in managing anxiety in outpatient dental surgical care, emphasizing the value of customized and specific music types, particularly classical music.

## Figures and Tables

**Table 1:** General characteristics of all the included studies.

Authors & Year	Country	Study Design	Comparison	Sample Size	Age (Mean ± SD or Range)	Age Group	Dental Procedure	Outcome Tool	Music Selection	Modified Jadad Scores
Abbasi 2021 [44]	Pakistan	RCT	Video “Little lovely dentist” Dental songs Tell–show–do Control	40 40 40 40	6.80 ± 2.10 years 8.15 ± 2.27 years 7.50 ± 2.30 years 7.27 ± 1.68 years	Children	Dental prophylaxis treatments	FIS	Author	5
Aitken 2002 [39]	USA	CT	Upbeat(folk) music Relaxing(instrumental) music Control	15 15 15	65.0 ± 8.0 months 67.7 ± 12 months 61.5 ± 11 months	Children	Restorative dentistry with local anesthesia	VPT, MCAS, NCBRS	Author	3.5
Bhusari 2023 [33]	India	RCT	Monaural beats Binaural auditory beats Control	15 15 15	9.47 ± 2.13 years 8.80 ± 2.08 years 8.60 ± 2.59 years	Children	Restoration of carious teeth	VPT	Author	5.5
Dixit 2020 [30]	India	RCT	Music (instrumental) therapy Bach flower therapy Control	40 40 40	62.8 ± 1.3 months 58.4 ± 1.3 months 63.0 ± 1.4 months	Children	Oral prophylaxis and fluoride treatment	FIS, NCBRS	Author	5.5
Gulnahar 2020 [34]	Turkey	RCT	Turkish music Classical music Soft rock (instrumental) music Control	20 20 20 20	51.0 ± 9.1 years 57.1 ± 16.6 years 48.0 ± 16.4 years 47.6 ± 13.3 years	Adult	Dental implant surgery with local anesthesia	CDAS	Author	3
Gupta 2017 [28]	India	CT	Upbeat music (folk) Relaxing music (instrumental) Control	20 20 20	3–7 years	Children	Restorative treatment with local anesthesia	VPT	Author	2.5
James 2021 [32]	India	CT	Aromatherapy Music distraction Control	50 50 50	6–8 years	Children	Restorative treatment	VPT, FIS	Author	2
Janthasila 2023 [45]	Thailand	RCT	Music therapy Aromatherapy Music therapy+ aromatherapy Control	33 31 32 32	11.0 ± 0.83 years 10.94 ± 0.89 years 10.88 ± 0.87 years 11.0 ± 0.88 years	Children	Coating services with Clinpro Sealant Refill	FIS, CFSS-DS	Patient	5
Karapicak 2023 [36]	Turkey	RCT	Music Control	35 35	24.2 ± 5.9 years 25.5 ± 7.4 years	Adult	Restoration of posterior occlusal dental caries with local anesthesia	MDAS	Patient	5.5
Khandelwal 2019 [29]	India	RCT	Audio distraction Audiovisual distraction (chair) Audiovisual distraction (ceiling) Control	20 20 20 20	4–10 years	Children	Screening, prophylaxis, restoration, extraction with local anesthesia	VPT, RMS-PS	Author	3
Kim 2011 [42]	Korea	RCT	Music treatment Control	106 113	Older than 18 years	Adult	Impacted mandibular third molar extraction with local anesthesia	CDAS	Patient	4
Kupeli 2020 [35]	Turkey	RCT	Turkish music Classical music Soft rock (instrumental) music Control	20 20 20 20	25.4 ± 4.2 years 24.9 ± 4.6 years 22.3 ± 3.8 years 23.8 ± 5.3 years	Adult	Third molar electively extracted with local anesthesia	CDAS	Author	3
Lahmann 2008 [40]	Germany	RCT	Brief relaxation Music distraction Control	29 28 30	37.1 ± 10.1 years 32.7 ± 12.2 years 43.7 ± 13.0 years	Adult	Restorative treatment for simple caries	STAI, HAQ	Patient	5
Lai 2008 [41]	Taiwan	RCT	Soothing piano (classical) music Control	22 22	47.4 ± 11.3 years	Adult	Root canal treatment	STAI	Patient	5.5
Marwah 2005 [23]	India	RCT	Instrumental music Nursery rhymes music Control	10 10 20	4–8 years	Children	Screening, prophylaxis, restoration, extraction	VPT, VCRS	Patient	2
Navit 2015 [26]	India	RCT	Instrumental music Musical nursery rhymes Movie songs Audio stories Control	30 30 30 30 30	6–12 years	Children	Screening, prophylaxis, restoration, extraction with local anesthesia	VPT, VCRS	Author	3
Nuvvula 2015 [27]	India	RCT	Audio (music) distraction Audiovisual distraction (3D video glasses) Control	30 30 30	8.40 ± 1.1 years 8.23 ± 1.1 years 8.67 ± 1.6 years	Children	Pulp therapies in primary first and second molars with local anesthesia	MCDAS(f)	Patient	5.5
Padawe 2023 [22]	India	RCT	Binaural beats Control	60 60	5.98 ± 2.04 years 5.76 ± 1.69 years	Children	Fluoride application, scaling, extraction, root canal treatment	VPT	Author	5.5
Pande 2020 [31]	India	RCT	Audio distraction Audiovisual distraction (virtual reality) Mobile phone game distraction Tell–show–do (control)	15 15 15 15	5–8 years	Children	Restoration of carious teeth	FIS	Patient	4.5
Pellicer 2024 [38]	Spain	RCT	Baroque music Classical music Control	26 27 25	45.7 ± 15.2 years	Adult	Immediate post-extraction implants with local anesthesia	MDAS	Author	6
Prabhakar 2007 [24]	India	CT	Audio distraction Audiovisual distraction Control	20 20 20	4–8 years	Children	Screening, prophylaxis, restoration, extraction with local anesthesia	VPT, VCRS	Author	1
Singh 2014 [25]	India	RCT	Various music Control	30 30	6–12 years	Children	Extraction	VPT	Patient	2
Sorribes 2023 [37]	Spain	RCT	Music therapy Virtual reality (VR) Control	91 93 91	30.12 ± 9.25 years 27.98 ± 8.75 years 29.70 ± 9.79 years	Adult	Extraction of impacted third molars with local anesthesia	STAI	Author	5.5
Yamashita 2019 [43]	Japan	RCT	Classical music Control	17 17	27.47 ± 6.52 years 27.94 ± 6.05 years	Adult	Extraction of an impacted mandibular third molar with local anesthesia	STAI, MDAS	Author	5

Note: CT, controlled trial; RCT, randomized controlled trial; VPT, Venham picture test; MCAS, Modified Corah Anxiety Scale; NCBRS, North Carolina Behavior Rating Scale; VCRS, Venham’s Clinical Anxiety Rating Scale; STAI, State–Trait Anxiety Inventory; HAQ, Hierarchical Anxiety Questionnaire; CDAS, Corah’s Dental Anxiety Scale; MCDAS(f), Modified Child Dental Anxiety Scale, faces version; MDAS, Modified Dental Anxiety Scale; RMS-PS, Raghavendra, Madhuri, and Sujata (RMS) Pictorial Scale; FIS, Facial Image Scale; CFSS-DS, Children’s Fear Survey Schedule—Dental Subscale.

**Table 2:** Modified Jadad scores for all the included studies.

Included Studies (First Author, Year)	D1	D2	D3	D4	D5	D6	D7	D8	Total
Abbasi 2021 [44]	1	1	0	0	1	1	0	1	5
Atiken 2002 [39]	0	0	0.5	1	0	1	0	1	3.5
Bhusari 2023 [33]	1	1	0.5	1	0	1	0	1	5.5
Dixit 2020 [30]	1	1	0.5	1	0	1	0	1	5.5
Gulnahar 2020 [34]	1	0	0	0	0	1	0	1	3
Gupta 2017 [28]	0	0	0.5	1	0	0	0	1	2.5
James 2021 [32]	0	0	0	0	0	1	0	1	2
Janthasila 2023 [45]	1	1	0	0	1	1	0	1	5
Karapicak 2023 [36]	1	1	0.5	0	1	1	0	1	5.5
Khandelwal 2019 [29]	1	0	0	0	0	1	0	1	3
Kim 2011 [42]	1	0	0	0	1	1	0	1	4
Kupeli 2020 [35]	1	0	0	0	0	1	0	1	3
Lahmann 2008 [40]	1	1	0	0	1	1	0	1	5
Lai 2008 [41]	1	1	0.5	0	1	1	0	1	5.5
Marwah 2005 [23]	1	0	0	0	0	0	0	1	2
Navit 2015 [26]	1	0	0	0	0	1	0	1	3
Nuvvula 2015 [27]	1	1	0.5	0	1	1	0	1	5.5
Padawe 2023 [22]	1	1	0.5	1	0	1	0	1	5.5
Pande 2020 [31]	1	1	0.5	0	0	1	0	1	4.5
Pellicer 2024 [38]	1	0	1	1	1	1	0	1	6
Prabhakar 2007 [24]	0	0	0	0	0	1	0	0	1
Singh 2014 [25]	1	0	0	0	0	0	0	1	2
Sorribes 2023 [37]	1	1	0.5	0	1	1	0	1	5.5
Yamashita 2019 [43]	1	1	0	0	1	1	0	1	5
								Average	4.1

The total score for each article ranged from 0 to 8; a score of 7 or 8 was considered to be of high quality, 4–6 of moderate quality, and 1–3 of low quality. D1: Was the study described as randomized? (Yes: +1/No: 0). D2: Was the method of randomization appropriate? (Yes: +1/No: −1/Not described: 0). D3: Was the study described as blinding? (double-blind: +1; single-blind: +0.5; No: 0). D4: Was the method of blinding appropriate? (Yes: +1/No: −1/Not described: 0). D5: Was there a description of withdrawals and dropouts? (Yes: +1/No: 0). D6: Was there a clear description of the inclusion and exclusion criteria? (Yes: +1/No: 0). D7: Was the method used to assess adverse effects described? (Yes: +1/No: 0). D8: Were the methods of statistical analysis described? (Yes: +1/No: 0).

**Table 3:** Meta-analysis of music and audiovisual intervention on anxiety during four dental visits.

Subgroup	*k*	Effect Size (SMD)	95% Confidence Interval	*p*
Anxiety Score				
First visit (screening)	3	0.44	0.03 to 0.85	0.040
Second visit (oral prophylaxis)	3	0.37	−0.04 to 0.78	0.070
Third visit (restoration)	3	0.33	−0.08 to 0.74	0.110
Fourth visit (extraction with LA)	3	0.70	0.28 to 1.12	0.001

Note: *k*, number studies; SMD, standardized mean difference; SMD values > 0 indicating less effective outcomes in the music group.

**Table 4:** Certainty assessment using the GRADE approach.

Certainty Assessment (GRADE Approach)											
Outcome	No. of Trials	Study Design	Risk of Bias	Inconsistency	Indirectness	Imprecision	Other	Number of Patients (*n*)		Effect Estimate (95% CI)	Quality of Evidence
								Music	Control		
Dental anxiety	24	RCTs	Serious *	Serious **	Not serious	Not serious	None	1020	810	SMD −0.61 (−0.89 to −0.32)	⨁⨁○○
											Low
Dental anxiety about invasive procedures under LA	16	RCTs	Serious *	Serious **	Not serious	Not serious	None	724	528	SMD −0.50 (−0.80 to −0.21)	⨁⨁○○
											Low

GRADE, Grading of Recommendations, Assessment, Development, and Evaluations; LA, local anesthesia; RCT, randomized controlled trials; CI, confidence interval; SMD, standard mean difference. * The modified Jadad score of included articles indicated low-to-moderate quality. ** Substantial heterogeneity (I square > 50%).

## Data Availability

The data relevant to this study can be found within the article and the Supplementary Materials file.

## Supplementary Materials

The following supporting information can be downloaded at https://www.mdpi.com/article/10.3390/jcm13216491/s1, Table S1: PRISMA 2020 Checklist; Table S2: Excluded studies and reasons; Table S3: The GRADE assessment for all outcomes; Figure S1: Risk of Bias assessment for included studies; Figure S2: Funnel plots of all outcomes.
